# Non-invasive prediction of olfactory recovery in severe CRSwNP: a cross-compartment translational nomogram based on secretory IL-17A

**DOI:** 10.3389/falgy.2026.1978136

**Published:** 2026-10-14

**Authors:** Hangtian Zhang, Linlu Wang, Tong Lu, Yilin Hou, Yi Wei, Wenhao Huang, Shaolin Liu, Yanran Huang, Zhaofeng Xu, Jian Li, Weiping Wen, Hua Zhong

**Affiliations:** 1 Department of Otorhinolaryngology, The First Affiliated Hospital of Sun Yat-sen University, Guangzhou, Guangdong, China; 2 Guangdong Engineering Research Center of Otorhinolaryngology Diagnosis and Treatment, Guangzhou, Guangdong, China; 3 Otorhinolaryngology Institute of Sun Yat-en University, Guangzhou, Guangdong, China; 4 Guangzhou Key Laboratory of Otorhinolaryngology, Guangzhou, Guangdong, China; 5 Department of Otolaryngology-Head and Neck Surgery, The Third Affiliated Hospital of SunYat-Sen University, Guangzhou, China; 6 Department of Allergy, The First Affiliated Hospital of Sun Yat-sen University, Otorhinolaryngology Institute of Sun Yat-sen University, Guangzhou, Guangdong, China; 7 Department of Otorhinolaryngology, Peking University Shenzhen Hospital, Shenzhen Peking University-the Hong Kong University of Science and Technology Medical Center, Shenzhen, Guangdong, China; 8 Guangxi Hospital Division of the First Affiliated Hospital, Sun Yat-sen University, Nanning, China; 9 Department of Otolaryngology, The Seventh Affiliated Hospital of Sun Yat-sen University, Guangzhou, Guangdong, China

**Keywords:** anosmia, biomarkers, machine learning, nasal mucosa, Type 2 inflammation

## Abstract

**Background:**

Predicting olfactory recovery after surgery for severe chronic rhinosinusitis with nasal polyps (CRSwNP) remains challenging. To overcome the reliance on invasive tissue biopsies, this study aimed to identify non-invasive liquid biopsy biomarkers reflecting the deep mucosal inflammatory microenvironment, ultimately developing a point-of-care prognostic model.

**Methods:**

A “tissue-to-secretion” dual-cohort validation strategy was employed. A Random Forest (RF) algorithm screened deep-tissue proteomes (Luminex) in a discovery cohort (*N* = 67). An independent RF model evaluated non-invasive nasal secretions (Olink) in a validation cohort (*N* = 62). Bidirectionally convergent targets were assessed for continuous dose-response relationships with absolute olfactory improvement [*Δ*(visual analog scale)VAS] score at 6 months. A clinical-molecular nomogram was developed and internally validated using 1,000 bootstrap resamples.

**Results:**

The tissue-based RF identified interleukin (IL)-17A as a core prognostic candidate. The independent secretion-based RF model also prioritized secretory IL-17A, achieving bidirectional convergence. While canonical Type 2 cytokines (IL-5, IL-13) lost predictive significance in secretions (*P* > 0.05), secretory IL-17A demonstrated a robust dose-response correlation (r = 0.334, *P* = 0.0003) and emerged as an independent protective factor for olfactory recovery ([odds ratio] OR = 1.408, 95% [confidence interval] CI: 1.03–2.03). The joint nomogram, seamlessly integrating baseline VAS and secretory IL-17A, yielded an optimism-corrected (area under the receiver operating characteristic curve) AUC of 0.793 (95% CI: [0.6908–0.9079]) with adequate calibration and substantial net clinical benefit.

**Conclusions:**

Through cross-compartment translation, we identified secretory IL-17A as a robust, non-invasive indicator of mucosal plasticity and olfactory reversibility. The derived joint nomogram provides an actionable framework for personalized pre-operative counseling and risk stratification in severe CRSwNP.

## 1 Introduction

Olfactory dysfunction remains one of the most debilitating and recalcitrant symptoms in patients with severe chronic rhinosinusitis with nasal polyps (CRSwNP) (1–4). While functional endoscopic sinus surgery (FESS) effectively alleviates mechanical obstruction and rhinorrhea, the recovery of olfactory function is highly unpredictable (5–7). A substantial subset of patients with severe disease experiences persistent olfactory loss even after successful surgical intervention, severely impairing their quality of life (8). Currently, clinicians face a significant dilemma: the lack of a reliable, objective prognostic tool to accurately stratify patients who will regain olfaction versus those who will remain refractory, which hinders personalized pre-operative counseling and timely medical interventions.

Conventionally, severe mucosal inflammation and subsequent olfactory neuroepithelial damage have been attributed to an overwhelming, homogenous Type 2 inflammatory burden [e.g., (interleukin) IL-4, IL-5, and IL-13] (9–12). However, evaluating Type 2 signatures alone often fails to fully explain the divergent trajectories of post-operative olfactory recovery (13, 14). Recently, the concept of a “mixed-endotype” (the co-existence of Type 1 or Type 3 neutrophilic inflammation alongside Type 2) has emerged as a crucial driver of severe polypoid remodeling, particularly in Asian cohorts (15–17). Despite this, the precise roles of these non-Type 2 mediators, particularly the IL-17 cytokine family, in determining olfactory reversibility remain poorly characterized and highly controversial (18, 19).

Mechanistically, exploring these deep inflammatory profiles has traditionally relied on invasive tissue biopsies, which are impractical for routine pre-operative screening or dynamic outpatient monitoring (20, 21). To circumvent this, there is an urgent need for non-invasive “liquid biopsy” alternatives. Nasal secretions, which directly bathe the olfactory cleft, offer an ideal biological window. Emerging evidence highlights that secretory proteins, particularly local cytokines and chemokines, can sensitively mirror the neuroepithelial microenvironment and hold promising diagnostic and prognostic value for olfactory disorders (22). However, the precise spatial mapping between deep-tissue mixed endotypes and superficial secretory phenotypes remains largely unknown. A systematic cross-compartment pipeline to filter bulk-tissue background noise and identify robust secretory surrogates is still lacking (23, 24).

To bridge this critical translational gap, the present study employed a novel “tissue-to-secretion” dual-cohort strategy (Figure 1). We first utilized a machine learning algorithm in a deep-tissue discovery cohort to outline the core inflammatory network. Subsequently, we stringently filtered these targets through an independent secretory validation cohort to identify the optimal non-invasive predictor. Ultimately, we aimed to develop and internally validate a clinical-molecular nomogram (25), integrating point-of-care secretory profiling with patient-reported severity, to provide a highly accurate and personalized prognostic framework for olfactory recovery in severe CRSwNP.

**Figure 1:**
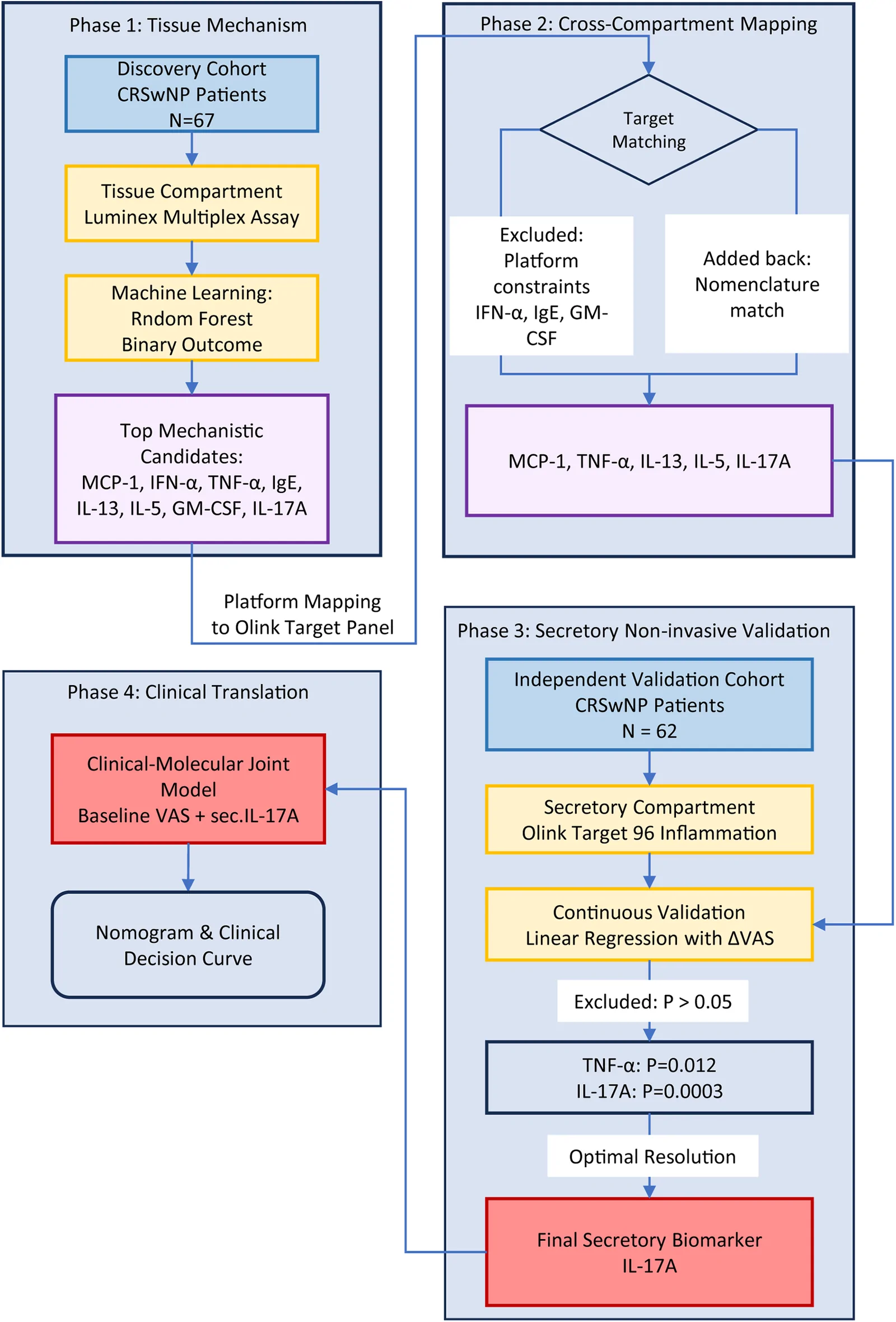
Study design and the “tissue-to-secretion” cross-compartment translational workflow.

## 2 Method

### 2.1 Study design and patient cohorts

This study was approved by the Ethics Committee on Scientific Research of the First Affiliated Hospital of Sun Yat-sen University (No. 2023–211). All participants provided written informed consent before the surgery. All diagnosis follow the European Position Paper on Sinusitis and Nasal Polyps guidelines (2). To ensure a well-defined and homogenous cohort, strict clinical criteria were applied. The inclusion criteria were: (1) a confirmed diagnosis of CRSwNP; and (2) patients scheduled to undergo functional endoscopic sinus surgery (FESS).The exclusion criteria were: (1) presence of antrochoanal polyps, cystic fibrosis, allergic fungal sinusitis, or unilateral NPs; (2) existence of malignant tumors, severe systemic chronic diseases, or infectious diseases; (3) autoimmune diseases and other systemic immune disorders; (4) long-term use of immunosuppressive medication; (5) age less than 18 or greater than 75 years; and (6) use of systemic steroids (e.g., oral corticosteroids), antibiotics, or nonsteroidal anti-inflammatory drugs for at least 4 weeks before surgery. All enrolled patients underwent functional endoscopic sinus surgery (FESS) and received long-term postoperative treatment with intranasal corticosteroids, antihistamines, and nasal irrigation. The patient's subjective olfactory function was assessed using a visual analog scale (VAS), with a score of 10 indicating anosmia. Patients were followed-up for 6 months after surgery, with an improvement of olfaction defined as an increase of ≥ 3 points in the postoperative subjective VAS score for olfaction (2).

The sample size was determined by the availability of high-quality, completely documented cases within our departmental clinical practice registry. Given the exploratory nature of the initial high-dimensional proteomic screening, a formal *a priori* sample size calculation was not strictly performed; however, the enrolled sample sizes of our dual cohorts are highly comparable to, and often exceed, those of similar exploratory biomarker discovery studies in the field of rhinology. To ensure the highest fidelity of the molecular mapping, a rigorous data filtering process was applied and patients with incomplete pre-operative clinical baseline records, degraded biospecimens, or lost to follow-up at 6 months were excluded. Consequently, the final analyzed cohorts (*N* = 67 for discovery and *N* = 62 for validation) contained zero missing data across the core predictive variables and primary clinical endpoints utilized for modeling (e.g., biomarker profiles, pre-operative VAS, and follow-up VAS). While minor missing data existed in certain non-modeled baseline characteristics, the completeness of the core predictors precluded the need for any multiple imputation techniques during nomogram construction.

### 2.2 Clinical endpoints and olfactory assessment

Patient-reported olfactory and gustatory function was evaluated using a VAS, ranging from 0 (no impairment) to 10 (total loss of function). Baseline VAS score was recorded pre-operatively, and follow-up VAS score was assessed at 6 months post-operation. The primary endpoint of this study was a “favorable olfactory recovery,” which was defined as a composite clinical outcome: an absolute post-operative improvement in VAS (*Δ*VAS) score of ≥ 3, or a final post-operative 6-month VAS score of ≤ 3 (2).

### 2.3 Biospecimen collection and protein profiling

The tissue-level protein expression data were obtained from a prospective institutional cohort study. Briefly, nasal polyp tissues were collected during endoscopic sinus surgery (ESS). For tissue processing, a total of 0.1 g of homogenized nasal polyp tissue was dissolved in 1 mL of 0.9% NaCl solution. The homogenate was subsequently centrifuged at 1,500 × g for 10 min to obtain the tissue lysates. The expression levels of core inflammatory biomarkers within the lysates were then quantified using a multiplex Luminex platform (Luminex 200 system, R&D Systems). All samples underwent normalization for total protein concentration to ensure inter-group comparability.

The secretory protein profiles used for model validation were derived from a previously described non-invasive liquid biopsy cohort (26). In brief, nasal fluids (NFs) were collected by inserting a synthetic absorption device made of polyester fiber into each nostril, which was left in place for 4 min on the day before surgery during pre-operative admission. The collected devices were subsequently centrifuged at 16,000  ×  g at 4° C for 10 min to elute the secretions. The supernatant was then harvested, and the targeted cytokine levels were quantified using high-sensitivity Proximity Extension Assay (PEA) technology via the Olink Target 96 Inflammation panel (Olink Proteomics, Uppsala, Sweden), as detailed in our previous methodology (26). In the current study, we performed a targeted cross-compartment re-analysis of this dataset, focusing on the dynamic relationship between secretory biomarkers and longitudinal olfactory outcomes (VAS scores at 1 and 6 months) to facilitate cross-compartment model integration.

### 2.4 Statistical software and environment

Continuous variables were presented as mean (standard deviation, SD) or median (interquartile range, IQR) depending on normality, and compared using Student's t-test or Mann–Whitney U test. Categorical variables were presented as counts (percentages) and compared using the chi-square or Fisher's exact test. All data processing, statistical analyses, and machine learning modeling were performed using R software (version 4.4.1, R Foundation for Statistical Computing). A 2-sided *P*-value < 0.05 was considered statistically significant.

### 2.5 Machine learning-driven biomarker selection

To examine high-dimensional tissue protein data, a Random Forest (RF) algorithm was implemented in the discovery cohort. Biomarker importance was ranked using the Mean Decrease Gini index, with candidates scoring >1.0 defined as top-tier targets for cross-compartment translation. These targets were cross-referenced with the Olink Target 96 panel and evaluated in the secretory validation cohort via Spearman's correlation against absolute olfactory improvement (*Δ*VAS). Concurrently, an independent RF model identified the top 10 secretion-native predictors. A dual-compartment cross-verification process, incorporating univariate regression and correlation analyses, was utilized to isolate the most robust, platform-independent secretory biomarkers for downstream modeling.

### 2.6 Nomogram construction and comprehensive evaluation

A multivariate logistic regression model was constructed incorporating the selected secretory biomarkers and pre-operative baseline VAS scores to determine independent predictive values. Based on these coefficients, a clinical-molecular nomogram was formulated using the rms package. The prognostic performance of the nomogram was comprehensively evaluated across 3 dimensions. Discrimination was assessed by the Area Under the Receiver Operating Characteristic Curve (AUC), with internal validation performed via 1,000 bootstrap resamples to yield an optimism-corrected AUC. Calibration curves, also generated via 1,000 bootstrap iterations, evaluated the concordance between predicted probabilities and actual observed recovery rates. Finally, Decision Curve Analysis (DCA) was conducted using the dcurves package to estimate the net clinical benefit across a continuum of threshold probabilities.

## 3 Results

### 3.1 Baseline characteristics of the discovery and validation cohorts

A total of 129 patients diagnosed with CRSwNP were enrolled in this study and stratified into 2 independent cohorts: the tissue discovery cohort (*N* = 67) for deep mucosal Luminex profiling, and the secretory validation cohort (*N* = 62) for non-invasive Olink targeted analysis. As summarized in Table 1, the 2 cohorts were well-matched regarding fundamental demographic and clinical features. Specifically, there were no statistically significant differences between the discovery and validation sets in terms of age, sex distribution, Lund-Mackay (LM) total scores, ethmoid-to-maxillary (E/M) ratios, olfactory cleft score, endoscopic Lund-Kennedy(LK) total score, peripheral blood neutrophil and eosinophil counts, the prevalence of comorbid asthma and allergic rhinitis (AR), and the total preoperative SNOT-22 score (all, *P* > 0.05). This robust baseline alignment ensures that the subsequent cross-compartment molecular mapping was not confounded by underlying demographic or clinical disparities.

**Table 1:** Baseline comparison between discovery group and validation group.

Variable	Level	Discovery (*n* = 67)	Validation (*n* = 62)	*p*-value	Test	Missing (%)
Demographic & Baseline Clinical Characteristics						
Age, mean (SD)		40.87 (14.69)	42.05 (14.53)	0.647	t	0
Gender, *n* (%)	Female	27 (40.3)	17 (27.4)	0.175	Fisher	0
	Male	40 (59.7)	45 (72.6)			
Baseline VAS score, mean (SD)		8.03 (1.60)	6.56 (3.89)	0.005	t	0
6-month Postoperative VAS score, mean (SD)		5.46 (3.43)	3.39 (3.84)	0.002	t	0
Asthma, *n* (%)	No	58 (86.6)	53 (85.5)	1	Fisher	0
	Yes	9 (13.4)	9 (14.5)			
Allergic Rhinitis (AR), *n* (%)	No	57 (85.1)	53 (85.5)	1	Fisher	0
	Yes	10 (14.9)	9 (14.5)			
Prior Sinus Surgery, *n* (%)	No	46 (68.7)	49 (79.0)	0.256	Fisher	0
	Yes	21 (31.3)	13 (21.0)			
Total IgE, median [IQR]		79.78 [22.36, 196.46]	88.31 [29.45, 174.35]	0.807	Nonnorm	29.5
House dust mite sensitization, *n* (%)	No	49 (73.1)	47 (75.8)	0.884	Fisher	0
	Yes	18 (26.9)	15 (24.2)			
Hematological Parameters, mean (SD)						
Neutrophil percentage (NEU%)		0.55 [0.49, 0.60]	0.53 [0.47, 0.59]	0.184	Nonnorm	0.8
Eosinophil percentage (EOS%)		0.04 [0.02, 0.08]	0.05 [0.02, 0.07]	0.608	Nonnorm	0.8
Absolute neutrophil count		3.57 [2.74, 4.38]	3.47 [2.60, 4.39]	0.856	Nonnorm	0.8
Absolute eosinophil count		0.27 [0.11, 0.52]	0.33 [0.19, 0.50]	0.548	Nonnorm	0.8
Lund-Mackay (LM) Sinus CT Score & Ethmoid/Maxillary Ratio						
LM total score, median [IQR]		15.00 [13.00, 19.00]	17.00 [12.00, 20.00]	0.695	Nonnorm	3.1
LM maxillary sinus score, median [IQR]		2.00 [2.00, 3.00]	2.00 [2.00, 3.00]	0.432	Nonnorm	3.1
LM anterior ethmoid score,median [IQR]		2.00 [2.00, 3.50]	3.00 [2.00, 4.00]	0.253	Nonnorm	3.1
LM posterior ethmoid score, median [IQR]		2.00 [2.00, 3.00]	2.00 [2.00, 4.00]	0.113	Nonnorm	3.1
LM sphenoid sinus score, median [IQR]		2.00 [1.00, 3.00]	2.00 [1.00, 3.00]	0.723	Nonnorm	3.1
LM frontal sinus score, median [IQR]		2.00 [2.00, 4.00]	3.00 [1.25, 4.00]	0.775	Nonnorm	3.1
LM ostiomeatal complex (OMC) score, median [IQR]		4.00 [4.00, 4.00]	4.00 [4.00, 4.00]	0.562	Nonnorm	3.1
Ethmoid/Maxillary Sinus Score Ratio (EM Ratio), median [IQR]		2.00 [2.00, 2.50]	2.00 [2.00, 3.00]	0.294	Nonnorm	3.1
Olfactory Cleft Scores (OCS), median [IQR]						
Total Olfactory Cleft Score (TOCS)		6.00 [3.00, 8.00]	6.00 [4.00, 8.00]	0.947	Nonnorm	3.1
Anterior Olfactory Cleft Score (AOCS)		3.00 [2.00, 4.00]	3.00 [2.00, 4.00]	0.688	Nonnorm	3.1
Posterior Olfactory Cleft Score (POCS)		3.00 [2.00, 4.00]	4.00 [3.00, 4.00]	0.484	Nonnorm	3.1
Right Anterior Olfactory Cleft Score (R_AOCS)		2.00 [1.00, 2.00]	2.00 [1.00, 2.00]	0.983	Nonnorm	3.1
Right Posterior Olfactory Cleft Score (R_POCS)		2.00 [1.00, 2.00]	2.00 [1.00, 2.00]	0.771	Nonnorm	3.1
Left Anterior Olfactory Cleft Score (L_AOCS)		2.00 [1.00, 2.00]	1.00 [0.00, 2.00]	0.616	Nonnorm	3.1
Left Posterior Olfactory Cleft Score (L_POCS)		2.00 [1.00, 2.00]	2.00 [1.00, 2.00]	0.315	Nonnorm	3.1
Preoperative Lund-Kennedy (LK) Endoscopic Scores, median [IQR]						
Preoperative LK total score		10.00 [8.00, 10.00]	10.00 [10.00, 12.00]	0.011	Nonnorm	0
Left (L) polyp score (preoperative)		2.00 [1.00, 2.00]	2.00 [2.00, 2.00]	0.384	Nonnorm	0
Left (L) discharge score (preoperative)		1.00 [1.00, 2.00]	2.00 [1.00, 2.00]	0.212	Nonnorm	0
Left (L) edema score (preoperative)		2.00 [1.00, 2.00]	2.00 [1.00, 2.00]	0.016	Nonnorm	0
Left (L) scarring score (preoperative)		0.00 [0.00, 0.00]	0.00 [0.00, 0.00]	N/A	Nonnorm	0
Left (L) crusting score (preoperative)		0.00 [0.00, 0.00]	0.00 [0.00, 0.00]	N/A	Nonnorm	0
Right (R) polyp score (preoperative)		2.00 [1.00, 2.00]	2.00 [2.00, 2.00]	0.111	Nonnorm	0
Right (R) discharge score (preoperative)		1.00 [1.00, 2.00]	2.00 [1.00, 2.00]	0.169	Nonnorm	0
Right (R) edema score (preoperative)		2.00 [1.00, 2.00]	2.00 [1.00, 2.00]	0.032	Nonnorm	0
Right (R) scarring score (preoperative)		0.00 [0.00, 0.00]	0.00 [0.00, 0.00]	N/A	Nonnorm	0
Right (R) crusting score (preoperative)		0.00 [0.00, 0.00]	0.00 [0.00, 0.00]	N/A	Nonnorm	0
Postoperative Lund-Kennedy (LK) Endoscopic Scores, median [IQR]						
Postoperative LK total score		2.00 [1.00, 6.00]	4.00 [2.00, 6.00]	0.161	Nonnorm	7
Left (L) polyp score (postoperative)		0.00 [0.00, 0.00]	0.00 [0.00, 0.00]	0.03	Nonnorm	7
Left (L) discharge score (postoperative)		1.00 [0.00, 2.00]	1.00 [0.00, 2.00]	0.847	Nonnorm	7
Left (L) edema score (postoperative)		1.00 [0.00, 1.00]	1.00 [0.00, 1.25]	0.483	Nonnorm	7
Left (L) scarring score (postoperative)		0.00 [0.00, 0.00]	0.00 [0.00, 0.00]	0.184	Nonnorm	7
Left (L) crusting score (postoperative)		0.00 [0.00, 0.00]	0.00 [0.00, 0.00]	0.077	Nonnorm	7
Right (R) polyp score (postoperative)		0.00 [0.00, 0.00]	0.00 [0.00, 0.00]	0.03	Nonnorm	7
Right (R) discharge score (postoperative)		1.00 [0.00, 2.00]	1.00 [0.00, 2.00]	0.45	Nonnorm	7
Right (R) edema score (postoperative)		1.00 [0.00, 1.00]	1.00 [0.00, 1.00]	0.237	Nonnorm	7
Right (R) scarring score (postoperative)		0.00 [0.00, 0.00]	0.00 [0.00, 0.00]	0.35	Nonnorm	7
Right (R) crusting score (postoperative)		0.00 [0.00, 0.00]	0.00 [0.00, 0.00]	0.867	Nonnorm	7
Preoperative SNOT-22 total score, mean (SD)		33.81 (17.38)	32.23 (19.77)	0.653	t	10.9

N/A: Statistical testing was not applicable due to zero variance across both cohorts.

Regarding olfactory severity, the discovery cohort exhibited a significantly higher baseline VAS score compared to the validation cohort (mean 8.03(1.60) vs. 6.56(3.89); mean difference = 1.47, 95% confidence interval [CI]: [0.41–2.52], *P* = 0.007). Correspondingly, the post-operative 6-month VAS scores were also higher in the discovery cohort (mean 5.46 vs. 3.39; mean difference = 2.07, 95% CI: [0.80–3.35], *P* = 0.002). Rather than indicating a selection bias, this variance in pre-operative severity reflects the typical real-world clinical heterogeneity (case-mix variation) inherent to different sampling compartments and recruitment phases. Importantly, validating prognostic biomarkers across cohorts with such distinct baseline severity profiles provides a highly rigorous evaluation of the model's spatial transportability and external generalizability, successfully avoiding the common pitfall of extreme-phenotype overfitting.

### 3.2 Tissue mechanistic discovery: A mixed-endotype inflammatory network

To elucidate the deep mucosal mechanisms dictating olfactory outcomes, we analyzed the tissue protein profiles from the Luminex discovery cohort (*N* = 67) utilizing a RF machine learning algorithm. By minimizing human selection bias, this non-linear classifier ranked the analytes based on their contribution to predictive accuracy, measured by the Mean Decrease Gini index (Figure 2).

**Figure 2:**
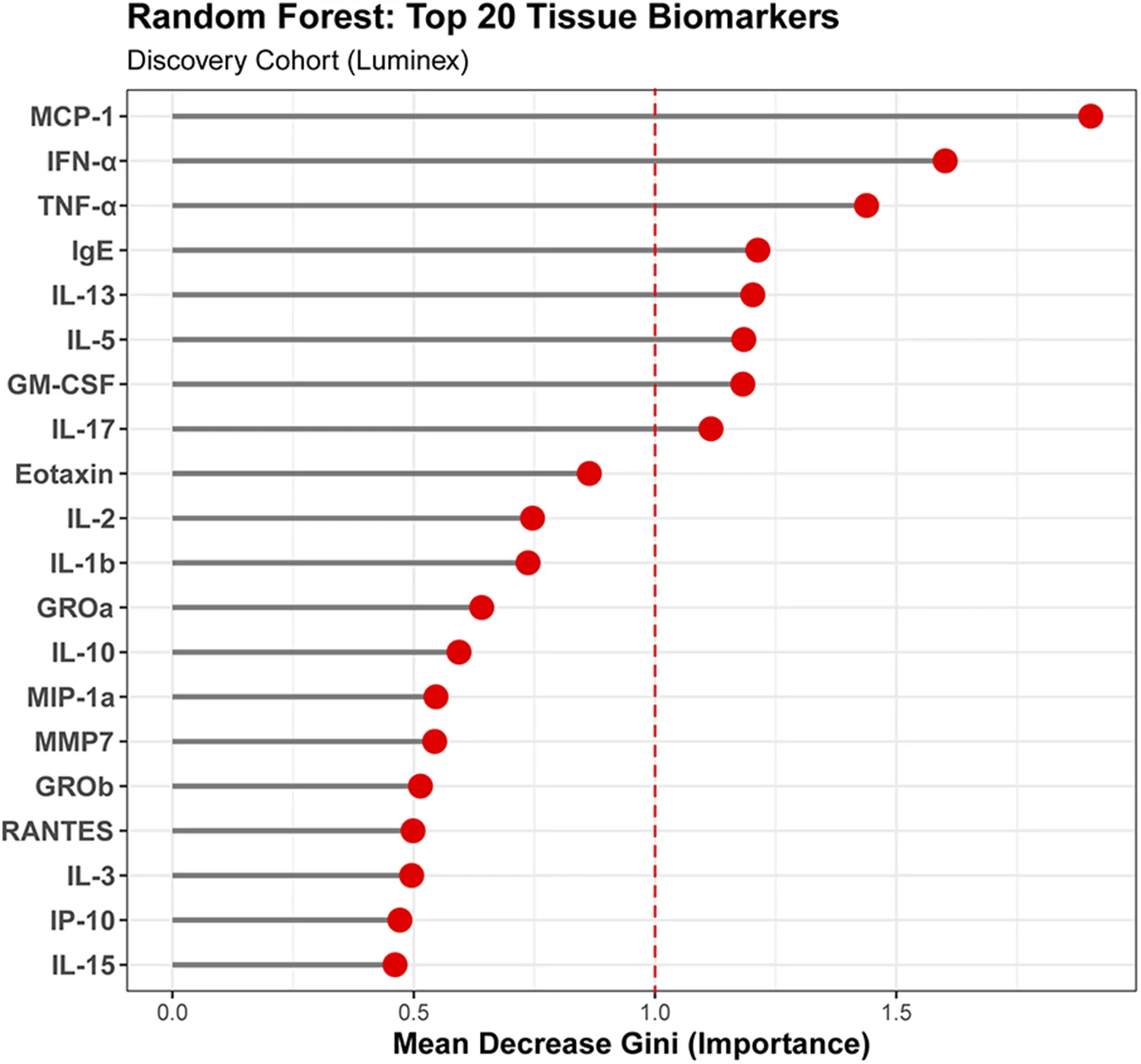
Identification of core inflammatory networks in the deep-tissue discovery cohort using the random forest algorithm.

The algorithm identified the top 20 most critical tissue mechanistic candidates. Notably, MCP-1 (Monocyte Chemoattractant Protein-1) emerged as the dominant overarching predictor, underscoring the profound involvement of macrophage recruitment in neuroepithelial dysfunction. Furthermore, the variable importance ranking revealed a compelling pathophysiological landscape: alongside canonical Type 2 inflammatory mediators (e.g., IL-13, IL-5, and IgE), several potent Type 1 and Type 3 neutrophilic cytokines [e.g., tumor necrosis factor (TNF)-α and IL-17] exhibited high predictive value.

Together, these tissue-level findings indicate that failure of post-operative olfactory recovery in CRSwNP is not exclusively driven by a homogenous Type 2 milieu. Instead, it is governed by a highly complex, mixed-endotype inflammatory storm. These top 20 candidates subsequently served as the evidence-based foundation for our cross-compartment translational mapping.

### 3.3 Cross-compartment targeted selection: From tissue discovery to secretory validation

To translate the deep-tissue mechanistic findings into a clinically applicable, non-invasive tool, we established a stringent cross-compartment filtering funnel (Figure 3). Initially, while the RF model delineated the broader important hierarchy of the top 20 tissue biomarkers (Figure 2), we applied the pre-defined empirical threshold (Mean Decrease Gini > 1.0) to strictly isolate the core pathogenic drivers. Consequently, 8 top-tier tissue candidates met this criterion. These 8 pivotal targets were then carried forward and cross-referenced with the secretory Olink Target 96 Inflammation panel. Due to platform limitations and specific target coverage, 3 candidates (IFN-α, IgE, and GM-CSF[granulocyte-macrophage colony stimulating factor]) were excluded. The remaining 5 candidates—MCP-1, TNF-α, IL-13, IL-5, and IL-17A—were successfully matched and quantified in the independent secretory validation cohort.

**Figure 3:**
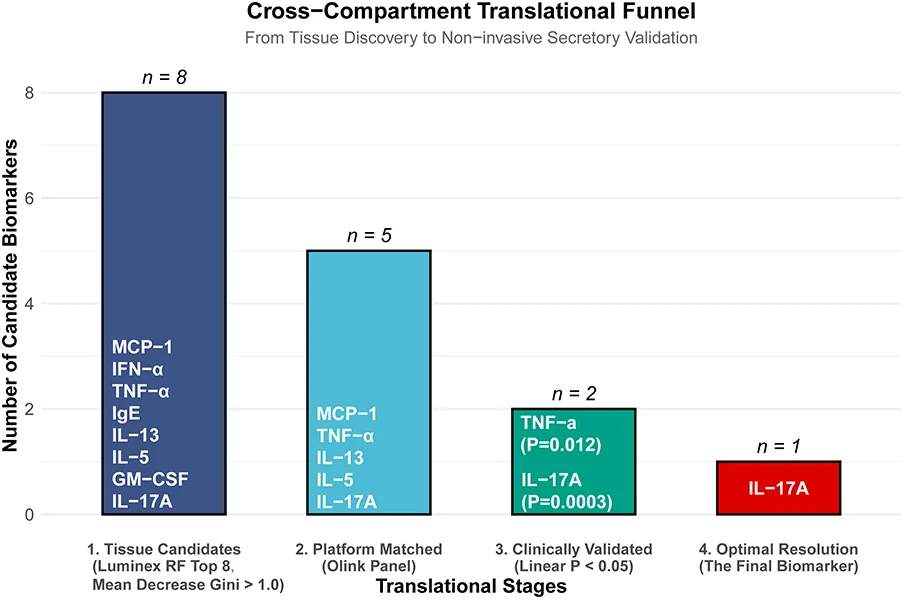
The cross-compartment targeted selection funnel.

Subsequently, we evaluated the continuous dose-response relationship between these matched secretory biomarkers and the absolute improvement in post-operative olfactory function (*Δ*VAS) (Table 2). Notably, canonical Type 2 cytokines (secretory IL-5 and IL-13) and the dominant tissue macrophage chemoattractant (secretory MCP-1) failed to reach statistical significance (all, *P* > 0.05). This loss of predictive power highlights the inherent discrepancy between the deep tissue milieu and the superficial secretory compartment, suggesting that bulk tissue signals cannot be blindly adopted as non-invasive surrogates.

**Table 2:** The cross-compartment targeted selection.

Luminex Rank	Tissue Biomarker	Olink Availability	Continuous Validation	Final Decision	Reason
1	MCP-1	Matched (sec.MCP-1)	0.540 (ns)	Excluded	Loss of cross-compartment predictive power
2	IFN-α	Not in Panel	N/A	Excluded	Platform constraint (Not in target panel)
3	TNF-α	Matched (sec.TNF)	0.0125 (*) r = 0.316 [0.07–0.52]	Excluded	Suboptimal predictive resolution vs. IL-17A
4	IgE	Not in Panel	N/A	Excluded	Platform constraint (Not in target panel)
5	IL-13	Matched (sec.IL13)	0.098 (ns)	Excluded	Lacks continuous dose-response significance
6	IL-5	Matched (sec.IL5)	0.262 (ns)	Excluded	Lacks continuous dose-response significance
7	GM-CSF	Not in Panel	N/A	Excluded	Platform constraint (Not in target panel)
8	IL-17A	Matched (sec.IL-17A)	0.0003 (***) r = 0.334 (0.42-0.81)	Selected	Strongest continuous dose-response power

In contrast, secretory biomarkers representing the Type 1 and Type 3 inflammatory axes demonstrated robust clinical relevance. While secretory TNF-α exhibited a moderate association with olfactory improvement (r = 0.316, 95% CI: [0.07- 0.52], *P* = 0.012), secretory IL-17A demonstrated a vastly superior predictive resolution, correlating with olfactory recovery at a significance level nearly 2 orders of magnitude stronger (r = 0.334, 95% CI: [0.42–0.81], *P* = 0.0003). To construct the most parsimonious and precise prognostic clinical tool, IL-17A was ultimately selected as the optimal non-invasive secretory biomarker for the joint predictive model.

### 3.4 Secretory IL-17A as an independent protective predictor

Having identified secretory IL-17A as the optimal non-invasive biomarker, we further characterized its prognostic utility regarding olfactory recovery. Initial continuous analysis revealed a highly significant positive correlation between the concentration of secretory IL-17A and the absolute improvement in olfactory visual analog scale scores (*Δ*VAS) at 6 months post-operation (Spearman r = 0.334, 95% CI: [0.42–0.81], *P* = 0.0003) (Figure 4A).

**Figure 4:**
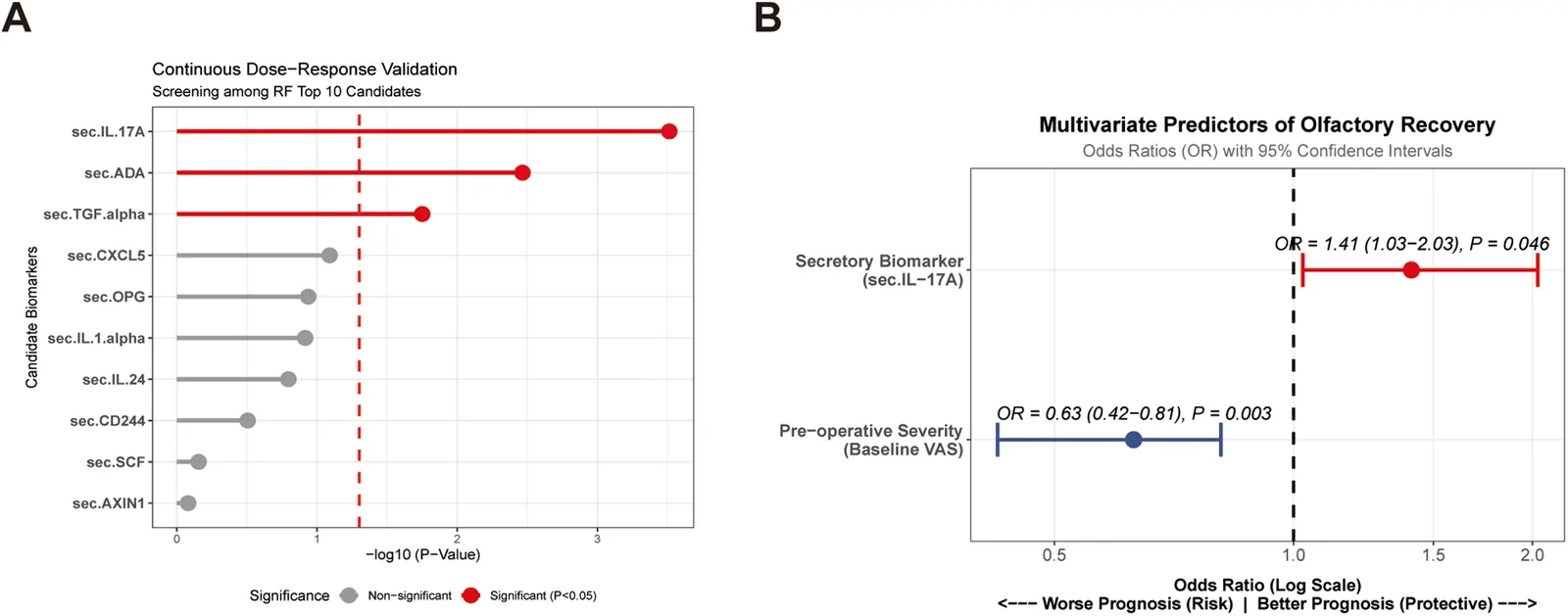
Independent prognostic value of secretory IL-17A for olfactory recovery. **(A)** Lollipop plot showing the initial continuous analysis, revealing a highly significant positive correlation (Spearman r = 0.334, 95% CI: [0.09–0.54], *P* = 0.0003) between secretory IL-17A concentrations and absolute olfactory improvement (*Δ*VAS) at 6 months post-operation. **(B)** Forest plot of the multivariate logistic regression model. Elevated secretory IL-17A emerged as a significant independent protective factor, while higher pre-operative baseline VAS acted as a significant risk factor for favorable recovery.

To rigorously evaluate its independent predictive value while accounting for clinical heterogeneity, we constructed a multivariate logistic regression model predicting the composite favorable recovery outcome (defined as an absolute *Δ*VAS improvement ≥ 3 or a final 6-month VAS ≤ 3). The model incorporated both the novel secretory biomarker (IL-17A) and the pre-operative disease severity (baseline VAS).

As illustrated in the forest plot (Figure 4B), baseline severity acted as a significant risk factor; higher pre-operative VAS scores independently decreased the likelihood of achieving the clinically meaningful recovery threshold (OR = 0.629, 95% CI: 0.46–0.86, *P* = 0.003). In stark contrast, elevated levels of secretory IL-17A emerged as a significant, independent protective prognostic factor for olfactory recovery (OR = 1.408, 95% CI: 1.03–2.03, *P* = 0.046). This intriguing finding indicates that within the severe CRSwNP microenvironment, the presence of an IL-17A-associated mixed inflammatory endotype (non-Type 2/Type 3 signature) may reflect a higher degree of neuroepithelial plasticity and post-operative reversibility compared to a homogenous, recalcitrant Type 2-dominant milieu.

### 3.5 Development and validation of the clinical-molecular nomogram

To facilitate personalized clinical decision-making, a non-invasive prognostic nomogram was constructed by integrating the identified independent predictors: the pre-operative clinical severity (baseline VAS) and the targeted secretory biomarker (IL-17A) (Figure 5). This visual tool allows rhinologists to intuitively estimate the probability of a favorable post-operative olfactory recovery for individual patients based on a simple combination of patient-reported outcomes and point-of-care secretory profiling.

**Figure 5:**
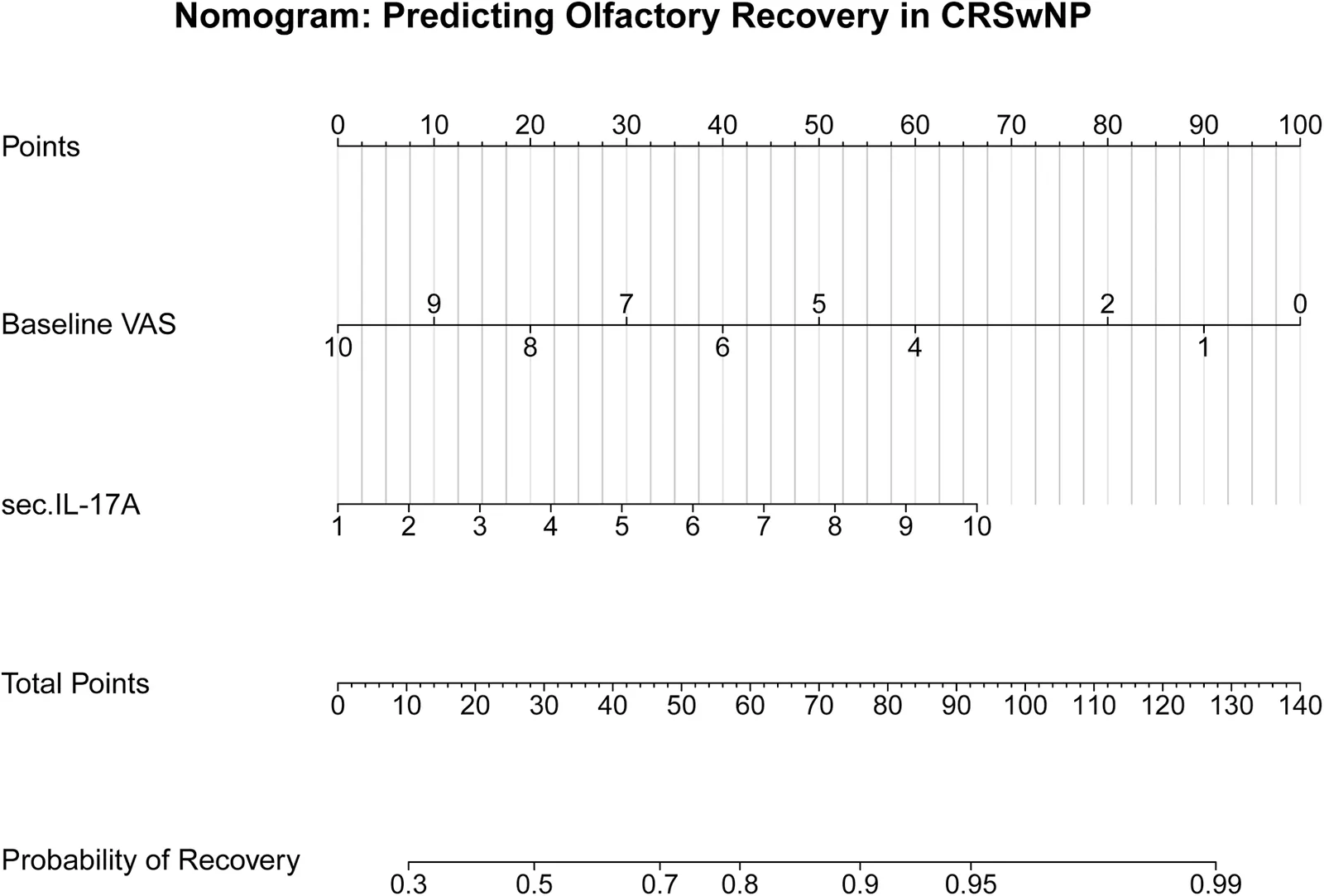
The clinical-molecular joint nomogram for predicting favorable post-operative olfactory recovery. Values for sec.IL-17A represent Normalized Protein Expression (NPX) on a log2 scale derived from the Olink PEA platform.

The predictive performance of the joint model was rigorously evaluated according to established guidelines. In terms of discrimination, the combined model achieved an excellent AUC of 0.805 (95% CI: 0.696–0.915) (Figure 6A). To robustly penalize potential overfitting and ensure internal validity, we subjected the model to 1,000 iterations of bootstrap resampling, which yielded a highly robust optimism-corrected AUC of 0.793(95% CI: [0.6908–0.9079]). Although classical severity markers, including peripheral blood eosinophil percentage, Lund-Mackay CT scores, and total olfactory cleft scores (TOCS), were evaluated in an expanded multivariate model, they did not exhibit independent predictive significance in the current cohort (Supplementary Table S1); thus, the nomogram was constructed strictly using independent predictors to ensure model parsimony.

**Figure 6:**
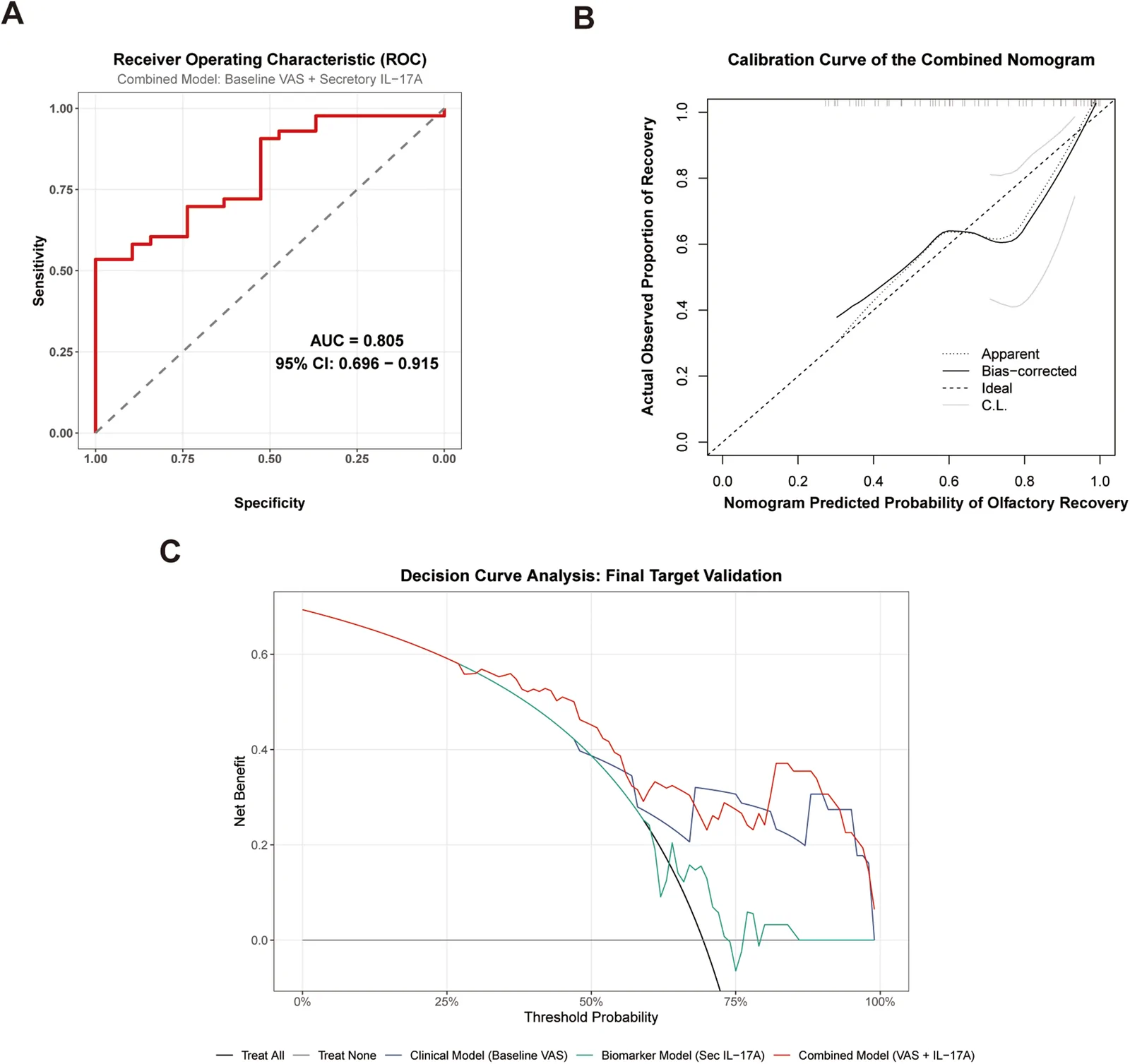
Comprehensive performance validation of the clinical-molecular nomogram. **(A)** Receiver Operating Characteristic (ROC) curve showing the excellent discriminative ability of the joint model, AUC = 0.805, 95% CI: [0.696–0.915]. (optimism-corrected AUC = 0.793, 95% CI: [0.6908–0.9079]). **(B)** Calibration curve evaluating the concordance between the nomogram-predicted probabilities and the actual observed olfactory recovery outcomes, generated via 1,000 bootstrap iterations. The ideal 45-degree dashed line represents perfect prediction, while the solid line represents the bias-corrected performance. The model demonstrates adequate overall calibration, although a moderate tendency to overestimate the recovery probability is observed in the intermediate-to-high range. **(C)** Decision Curve Analysis (DCA) indicating that the joint nomogram provides a substantially superior net clinical benefit compared to “treat-all”, “treat-none”, or baseline VAS-only strategies across a wide range of threshold probabilities.

Furthermore, the calibration curve demonstrated an acceptable concordance between the nomogram-predicted probabilities and the actual observed recovery outcomes, although the model exhibited a moderate tendency to overestimate the probability of recovery in the intermediate-to-high risk range (Figure 6B). This degree of variance is typical for cohorts of this size and indicates adequate internal validity. Finally, Decision Curve Analysis (DCA) was employed to evaluate real-world clinical utility. As depicted in Figure 6C, the clinical-molecular joint nomogram provided a substantially superior net clinical benefit across a broad, clinically relevant spectrum of threshold probabilities when compared to “treat-all” or “treat-none” default strategies, as well as a model relying solely on baseline VAS. Collectively, these validations underscore the nomogram as a precise and clinically valuable tool for pre-operative counseling and risk stratification in CRSwNP.

## 4 Discussion

Olfactory dysfunction remains one of the most debilitating and recalcitrant symptoms in patients with severe CRSwNP (2, 3, 27, 28), yet predicting post-operative olfactory recovery is severely hindered by the lack of reliable, non-invasive prognostic biomarkers (6). To bridge this critical clinical gap, the present study employed a novel “tissue-to-secretion” cross-compartment translational strategy, systematically mapping the complex deep mucosal inflammatory network to point-of-care liquid biopsy surrogates. By filtering out the profound “Type 2 noise” predominant in the bulk tissue milieu, we successfully identified and independently validated secretory IL-17A as a robust, protective prognostic factor for favorable olfactory outcomes. Integrating this targeted secretory biomarker with pre-operative clinical severity (baseline VAS), we developed and rigorously validated a clinical-molecular nomogram. This non-invasive tool demonstrated excellent predictive discrimination (optimism-corrected AUC = 0.793, 95% CI: [0.6908–0.9079]), adequate calibration, and substantial net clinical benefit, thereby providing rhinologists with a highly actionable and personalized framework for pre-operative counseling and risk stratification (29).

Perhaps the most biologically intriguing finding of our study is the counterintuitive, independent protective role of secretory IL-17A in post-operative olfactory recovery. Conventionally, IL-17A is recognized as a potent pro-inflammatory cytokine that disrupts epithelial tight junctions and drives neutrophilic infiltration (17, 18, 30). In CRSwNP, chronic olfactory dysfunction is classically attributed to an overwhelming, homogenous Type 2 inflammatory burden (e.g., IL-5, IL-13), which ultimately drives irreversible neuroepithelial remodeling, squamous metaplasia, and fibrotic entrapment of the olfactory cleft (9, 10, 31). However, our data suggest a critical paradigm shift: within an extreme, recalcitrant CRSwNP cohort, the robust presence of an IL-17A-mediated mixed endotype (Type 2/Type 3 co-signature) represents a fundamentally distinct mucosal state (12, 15, 16). We hypothesize that while a “pure,” end-stage Type 2 microenvironment leads to terminal and irreversible neural degradation (14), the acute epithelial disruption and active neutrophilic engagement orchestrated by IL-17A may reflect a higher degree of biological “plasticity” (32, 33). Consequently, following the surgical clearance of the inflammatory polypoid load and the administration of standard post-operative anti-inflammatory therapies, the neuroepithelium in these mixed-endotype patients retains a superior capacity for regeneration and functional reversibility (34). Thus, rather than merely acting as a destructive agent, elevated secretory IL-17A serves as a crucial surrogate marker for mucosal plasticity within a highly homogenous Type 2 disease spectrum.

Beyond these pathophysiological insights, the pronounced predictive discrepancy of IL-17A between the tissue and secretory compartments underscores a critical concept in spatial biology (13). While our Random Forest model highlighted IL-17A's interactive importance within the deep mucosal network, its standalone linear predictive capacity in bulk tissue homogenates was notably weak. This attenuation likely stems from the low signal-to-noise ratio inherent to whole-tissue analysis, where the independent prognostic signature of IL-17A is heavily masked by the overwhelming “Type 2 noise” and complex stromal cell interactions within the polypoid bed (20, 23). In stark contrast, mapping this biomarker to the superficial secretory compartment yielded a robust predictive resolution. We postulate that the presence of a high-concentration of IL-17A in nasal secretions does not merely reflect localized mucosal production; rather, it serves as a direct, functional readout of epithelial barrier collapse and tight junction disruption (22, 35). Therefore, the secretory compartment effectively acts as a biological filter, isolating the terminal pathogenic signals of neuroepithelial leakage from the convoluted deep-tissue inflammatory background, thereby validating the absolute necessity of our cross-compartment translational design.

Translating these spatial and biological insights into point-of-care practice, we developed a non-invasive clinical-molecular nomogram. The rationale for incorporating pre-operative symptom severity (baseline VAS) as a core variable is firmly supported by multiple large-cohort studies, which have consistently highlighted baseline olfactory function as a prognostic cornerstone. For instance, in a retrospective analysis of 791 patients with post-infectious olfactory dysfunction (PIOD), the severity of baseline anosmia, assessed via Sniffin' Sticks, was identified as a robust predictor for the lack of mid-term recovery (*P* < 0.001) (36). Similarly, other studies have corroborated that baseline psychophysical test scores represent the strongest independent predictors of eventual sensory restoration (37). However, relying solely on a single clinical parameter often yields limited predictive efficacy, necessitating the integration of multi-modal biomarkers. By synergizing this foundational patient-reported outcome with an objective molecular readout (secretory IL-17A), our visual nomogram provides rhinologists with a highly actionable framework to quantify individual probabilities of post-operative olfactory recovery. As demonstrated by the DCA, deploying this joint model yields a substantially higher net clinical benefit across a broad spectrum of threshold probabilities compared to default empirical strategies (38). Consequently, it facilitates personalized pre-operative counseling, empowering clinicians to preemptively identify refractory patients who might necessitate intensified perioperative medical adjuncts, such as targeted biologics or systemic corticosteroids, rather than relying solely on surgical intervention. Crucially, the methodological robustness of our model was rigorously challenged. In accordance with the TRIPOD (Transparent Reporting of a multivariable prediction model for Individual Prognosis or Diagnosis) guidelines, validating a predictive model in an independent cohort with a distinct case-mix variation provides a far more stringent evaluation of its transportability than validation in a perfectly matched dataset (25, 39). Furthermore, the overall acceptable concordance observed in our calibration curve suggests that the prognostic efficacy of our model is not fundamentally driven by extreme-phenotype overfitting, though the slight overestimation observed warrants cautious interpretation in mid-to-high probability stratifications.

Several limitations of the current study should be acknowledged. Firstly, while our joint model underwent rigorous internal validation utilizing bootstrap resampling, machine learning algorithms like Random Forest are inherently susceptible to overfitting, and our study lacks an independent external validation cohort. Consequently, this single-center design limits the immediate global generalizability of the nomogram. Future multicenter, prospective studies are warranted to externally validate its prognostic robustness. Secondly, our cross-compartment translational approach primarily establishes a potent prognostic association rather than direct biological causality. Subsequent *in vitro* and *in vivo* experimental models are required to definitively map the causal mechanistic link between IL-17A and olfactory neuroepithelial regeneration. Thirdly, although subjective VAS scoring is practical and validated for real-world clinical follow-up, future prospective multi-center studies incorporating objective psychophysical testing (e.g., Sniffin' Sticks) will provide valuable objective validation and strengthen the multidimensional evaluation of olfactory recovery.

In conclusion, our study successfully translates complex deep-tissue inflammatory mechanisms into a highly practical point-of-care tool through a stringent cross-compartment validation strategy. By filtering out the profound Type 2 background noise predominant in severe CRSwNP, we identified secretory IL-17A as a highly specific, independent protective predictor of olfactory recovery. This non-invasive framework empowers rhinologists to optimize personalized perioperative decision-making, ultimately improving the precise management of recalcitrant olfactory dysfunction.

## Data Availability

The datasets presented in this study can be found in online repositories. The names of the repository/repositories and accession number(s) can be found in the article/Supplementary Material.

## Supplementary material

SUPPLEMENTARY TABLE S1 Expanded multivariate logistic regression analysis for favorable post-operative olfactory recovery.

SUPPLEMENTARY FIGURE S1 Predictive capacity of tissue-derived IL-17 in the deep mucosal compartment ROC curve demonstrating the weak standalone linear predictive capacity of IL-17 in bulk tissue homogenates (AUC = 0.52), highlighting the heavy masking effect of the deep-tissue “Type 2 noise” and justifying the necessity of cross-compartment translation.
